# The Editor’s Salutation

**Published:** 1867-03

**Authors:** J. Adams Allen


THE EDITOR'S SALUTATION.—In resuming the editorial pen, laid down a little over three years' since with sensations of relief from high responsibilities and arduous labors, the undersigned admits a feeling of embarrassment in re-introducing himself to his journalistic audience. Unexpected events, some melancholy and some joyous, thrust the position upon him with a rapidity which yet disorders respiratory movements.

A considerable proportion of the present number of the JOURNAL had already passed through the hands of the compositors before this consummation was reached. Suffice it to say, that the new editor would not have again mounted the tripod had he not received the most satisfactory assurances of assistance, in filling the pages of the JOURNAL, from professional gentlemen, thoroughly competent both to interest and instruct.

On the part of the editor—neither zeal, time, nor expense shall be spared to maintain the JOURNAL in its proper position as the leading exponent of the profession in the North-west. Identified with that profession for the last twenty years, he does not deem it arrogance to assume that he knows much of its needs, and recognizes in full its claims to consideration. The JOURNAL will do its best to satisfy the former and secure appropriate acknowledgement of the latter.

For the better accomplishment of these ends, contributions are solicited from all who believe they have something of value to communicate.

The records of practical experience, whether in contending with individual isolated and rare cases, or with great and pervading epidemics, alike have interest to the reader.

The professional mind was never more active than now. Individuals and societies are working together for general advancement, under the sway of those broad and comprehensive views, which at once are necessary for the development and recognition of great truths, and not inconsistent with accurate and exhaustive study of the minutest details of science. To whatever it is believed will aid in this work, the pages of this journal will be opened with that catholicity of opinion which is, or should be, the crowning attribute of the profession.

To be the medium of communication between observers and thinkers; to foster the growth of organizations subsidiary to scientific investigation and instruction; to maintain legitimate medicine in its present proud superiority to all the petty and narrow "systems" which, from time immemorial, have striven to disgrace it by claiming affiliation with it—to be in the vanguard of those who are most earnestly attacking the domain of the unknown with determination to let in the light of knowledge to its dark and waste places—these will be among the prominent objects which the JOURNAL proposes to itself, and wherein it hopes to be fully sustained by the coöperation of its intelligent and enlightened readers.

J. ADAMS ALLEN.

